# Effects of combined non-invasive brain stimulation and brain-computer interface on upper extremity motor function after stroke: a systematic review and meta-analysis

**DOI:** 10.3389/fneur.2026.1993570

**Published:** 2026-10-19

**Authors:** Shutao Qiu, Li Zhang, Qiang Gao

**Affiliations:** 1 Rehabilitation Medicine Center and Institute of Rehabilitation Medicine, West China Hospital, Sichuan University, Chengdu, China; 2 Key Laboratory of Rehabilitation Medicine in Sichuan Province, West China Hospital, Sichuan University, Chengdu, China

**Keywords:** brain-computer interface, meta-analysis, non-invasive brain stimulation, stroke, systematic review, upper extremity

## Abstract

**Background:**

Non-invasive brain stimulation (NIBS) and brain-computer interface (BCI) training are both promising approaches for post-stroke upper extremity rehabilitation, and their combination has been hypothesized to produce synergistic effects. However, the efficacy of this combined strategy has not been systematically evaluated.

**Methods:**

We systematically searched EMBASE, PubMed, Web of Science, and the Cochrane Library from inception to October 2025 for randomized controlled trials evaluating NIBS combined with BCI in stroke rehabilitation. Mean differences (MDs) in pre- to post-intervention change scores and 95% confidence intervals (CIs) were pooled. Prespecified subgroup analyses were conducted to examine potential moderators.

**Results:**

Seven randomized controlled trials were included. Pooled analysis showed that NIBS combined with BCI did not provide superior immediate upper extremity motor recovery compared with BCI alone and was associated with lower FMA-UE improvement (MD = −3.67, 95% CI −5.32 to −2.01; I^2^ = 0%). Subgroup analysis suggested that this effect was mainly driven by studies of tDCS plus BCI, while no significant difference was observed for rTMS plus BCI. No significant long-term effect of tDCS plus BCI or stimulation-site difference for rTMS plus BCI was identified. However, the certainty of evidence ranged from moderate to very low, and sensitivity analyses suggested that the findings were unstable.

**Conclusion:**

Current evidence does not demonstrate a clear additional benefit of NIBS as an adjunct to BCI for upper extremity rehabilitation after stroke. Given the limited number of studies and the instability of the pooled result in sensitivity analysis, these findings should be interpreted cautiously. More rigorous and adequately powered trials are needed to determine whether optimized stimulation protocols or specific patient subgroups may benefit from this combined strategy.

**Systematic review registration:**

https://www.crd.york.ac.uk/PROSPERO/, identifier CRD420251151708.

## Introduction

Stroke remains one of the leading causes of death and long-term disability worldwide, imposing a substantial burden on patients, families, and healthcare systems (1, 2). Among stroke survivors, upper extremity motor impairment is particularly common and often persists beyond the acute phase, limiting independence in activities of daily living and restricting social participation (3, 4). Despite advances in conventional rehabilitation, recovery of upper extremity function remains challenging, and many patients achieve only partial improvement. Identifying interventions that can further enhance motor recovery therefore remains a major priority in stroke rehabilitation.

In recent years, neurotechnology-based interventions have attracted increasing attention as adjuncts to conventional therapy. Among these, non-invasive brain stimulation (NIBS) and brain-computer interface (BCI) are two approaches of particular interest. NIBS techniques, including transcranial magnetic stimulation (TMS), transcranial direct current stimulation (tDCS), and transcranial alternating current stimulation (tACS), can modulate cortical excitability and influence activity within motor networks (5–7). BCI, by translating brain signals into control commands for external devices or feedback systems, enables patients to engage in task-oriented training based on motor intention, thereby strengthening sensorimotor coupling and promoting use-dependent plasticity (8–11).

The rationale for combining NIBS with BCI is grounded in their potentially complementary mechanisms. NIBS may prime the central nervous system by altering cortical excitability, reducing maladaptive interhemispheric imbalance, and creating a more favorable neural state for motor learning. BCI, in turn, can provide contingent feedback linked to volitional brain activity and reinforce motor-related neural processes during training. From this perspective, the combination of NIBS and BCI may not simply represent the addition of two interventions, but rather a strategy to enhance neuroplasticity through coordinated modulation of cortical state and task-specific feedback (5, 12).

Although a growing number of clinical studies have investigated NIBS combined with BCI in patients with stroke, the findings have not been synthesized systematically (10). Existing reviews have typically focused on NIBS or BCI as separate interventions, whereas the effectiveness of their combined application remains unclear. In particular, whether different forms of NIBS confer distinct benefits when integrated with BCI, and whether stimulation applied to the ipsilesional or contralesional hemisphere yields different outcomes, has not been adequately established (13–15). These issues are clinically relevant, as they may influence both intervention design and patient selection.

Therefore, we performed a systematic review and meta-analysis to evaluate the efficacy of NIBS combined with BCI in stroke rehabilitation. Specifically, we compared NIBS plus BCI with BCI alone, with upper extremity motor function as the primary outcome. We also conducted subgroup analyses according to NIBS modality and explored the differential effects of ipsilesional and contralesional repetitive TMS (rTMS). By synthesizing the available evidence, this study aimed to clarify the therapeutic value of combining NIBS with BCI and to provide a basis for future clinical application and research.

## Methods

### Protocol and literature search

This systematic review and meta-analysis was conducted in accordance with the Cochrane Handbook for Systematic Reviews of Interventions and reported following the Preferred Reporting Items for Systematic Reviews and Meta-Analyses (PRISMA) guidelines (16, 17). The study protocol was prospectively registered in the International Prospective Register of Systematic Reviews (PROSPERO; registration number: CRD420251151708). A comprehensive literature search was performed in EMBASE, PubMed, Web of Science, and the Cochrane Library from database inception to October 25, 2025. The search strategy combined keywords and Medical Subject Headings (MeSH) terms related to “stroke,” “non-invasive brain stimulation,” and “brain-computer interface,” using Boolean operators (AND/OR). The full search strategies for all databases are provided in Supplementary Table S1.

### Eligibility criteria

Studies were selected according to the Participants, Intervention, Comparison, Outcomes, and Study design (PICOS) framework (18). Eligible studies met the following criteria: (1) participants were patients with clinically diagnosed ischemic or hemorrhagic stroke, irrespective of stroke stage (acute, subacute, or chronic); (2) the intervention involved combined NIBS and BCI therapy, defined as the delivery of both modalities within the same treatment program with the intention of producing a combined therapeutic effect. Concurrent delivery referred to NIBS and BCI being administered during the same treatment session or in an overlapping manner, whereas sequential delivery referred to the two modalities being delivered in a predefined temporal order within the same treatment session or treatment course. Eligible NIBS modalities included tDCS, rTMS, and theta burst stimulation (TBS), with no restrictions on stimulation protocols, BCI paradigms, or stimulation sites; (3) the comparison group received any active or inactive control condition, including BCI alone, sham stimulation, conventional rehabilitation, occupational therapy, usual care, or other therapies; (4) studies reported at least one prespecified stroke-related clinical outcome assessed at baseline and post-intervention or follow-up, including upper extremity motor function, functional independence, or safety outcomes; and (5) the study design was a randomized. Studies were excluded if they: (1) provided insufficient quantitative data for effect size estimation; (2) did not report extractable or imputable data in the published article; (3) had unavailable data after attempts to contact the corresponding authors; or (4) were reviews, case reports, conference abstracts, editorials, or other non-original studies.

### Study selection and data extraction

Two reviewers independently screened the titles, abstracts, and full texts of the retrieved records to determine study eligibility. Any disagreements were resolved through discussion, and, if necessary, by consultation with a third reviewer. Data were independently extracted by two reviewers using a standardized data extraction form, with discrepancies resolved by consensus or adjudication by a third reviewer. The following information was extracted from each study: (1) first author and year of publication; (2) sample size; (3) participant characteristics, including age and stroke stage/disease duration; (4) intervention details, including the combined therapy protocol, number of sessions, type of non-invasive brain stimulation, stimulation site, stimulation parameters, number of pulses per session (for rTMS), and total number of pulses per treatment (for rTMS); and (5) outcome data on efficacy and safety.

### Assessment of risk of bias and certainty of evidence

The methodological quality of the included studies was assessed using the 11-item Physiotherapy Evidence Database (PEDro) scale (19, 20). Although the first item is not included in the total score, it was recorded for completeness. PEDro scores were interpreted as follows: 9–10, excellent; 6–8, good; 4–5, fair; and ≤3, poor (18). Two reviewers independently performed the quality assessment, and any disagreements were resolved through discussion or consultation with a third reviewer. The certainty of evidence for each outcome was evaluated using the Grading of Recommendations Assessment, Development and Evaluation (GRADE) approach (19). The certainty of evidence was rated as high, moderate, low, or very low (21). The evidence could be downgraded based on the following domains: risk of bias, inconsistency, indirectness, imprecision, and publication bias (22). Inconsistency was considered when substantial heterogeneity was present (e.g., I^2^ > 50%).

### Data analysis

Meta-analysis was conducted using Review Manager (RevMan, version 5.4). Mean change scores from baseline to post-intervention or follow-up were used when available; otherwise, change scores and their standard deviations (SDs) were calculated from pre- and post-intervention/follow-up data based on Cochrane Handbook guidance. In multi-arm trials with a shared control group, relevant intervention arms were combined into a single comparison to avoid double counting. Heterogeneity was assessed using the chi-square test and the I^2^ statistic, with values of 25%, 50%, and 75% representing low, moderate, and high heterogeneity, respectively. Because clinical heterogeneity across studies was anticipated, pooled effect sizes were primarily estimated using a random-effects model. Subgroup analyses were performed, where appropriate, according to stroke stage/disease duration, and leave-one-out sensitivity analyses were used to examine the robustness of the pooled results.

## Results

### Study selection and characteristics

As shown in Figure 1, 555 records were identified through the initial search. After removal of 163 duplicates, 392 records were screened by title and abstract, and 217 were excluded. The full texts of 175 articles were assessed for eligibility, of which 164 were excluded. Multiple publications were derived from the same registered clinical trial (NCT01897025) (23–27). To avoid duplicate reporting, only Chew et al. (26) was included. One study was included only in the qualitative synthesis because the required data could not be extracted for quantitative analysis. Jia et al. (28) was a multi-arm trial with three eligible rTMS intervention arms sharing a common control group; for quantitative synthesis, these arms were combined into a single comparison. Ultimately, 7 studies were included in the systematic review.

**Figure 1:**
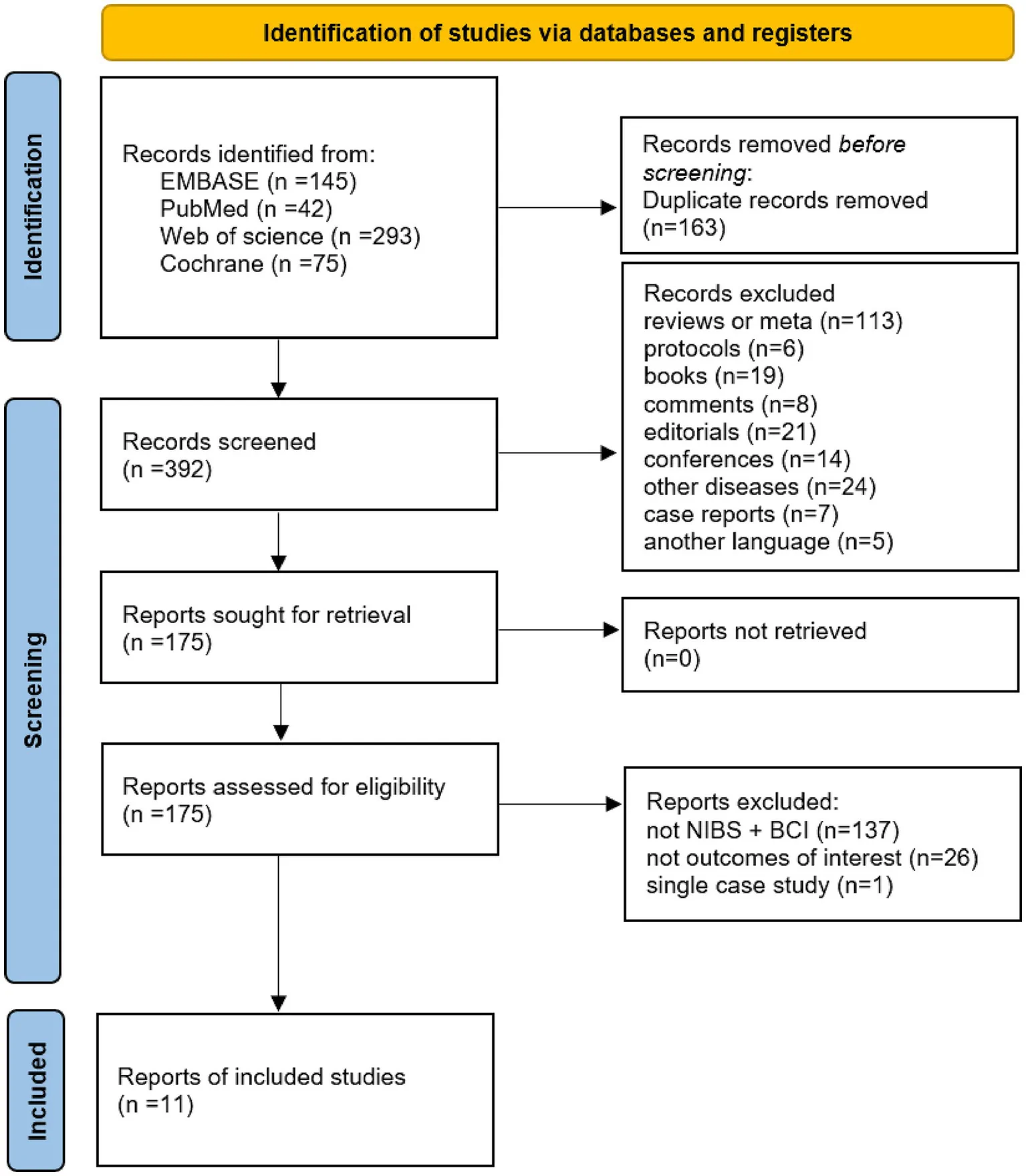
Preferred reporting items for systematic review and meta-analyses flow diagram of study selection.

The included studies enrolled patients in the subacute or chronic phase after stroke. In all studies, the experimental groups received non-invasive brain stimulation combined with BCI training, whereas the control groups received BCI training alone. The stimulation modalities included transcranial direct current stimulation and repetitive transcranial magnetic stimulation. The Fugl-Meyer Assessment for Upper Extremity (FMA-UE) was reported in all included studies and was used as the primary motor outcome for this review. Secondary outcomes included the Action Research Arm Test (ARAT), Motor Status Scale (MSS), functional assessments such as the Modified Barthel Index (MBI), cognitive assessments such as the Mini-Mental State Examination (MMSE), and neurophysiological or neuroimaging measures. Detailed study characteristics are presented in Table 1. ARAT, MSS, MBI, and MMSE were reported in only a limited number of studies. Because the available data were sparse and not sufficiently comparable, these outcomes were not meta-analyzed.

**Table 1:** Characteristics of the included studies.

Study	Country	Stage	*N*	Intervention	Control	Stimulation parameters	Outcomes
Kasashima-Shindo, 2015 (40)	Japan	Chronic stroke	18	tDCS + MI-BCI	MI-BCI	Cathodal tDCS over M1: Intensity 1 mA,10 min BCI: 45 min, 10 sessions over 2 weeks	FMA-UE, MAS, ERD
Hu (2021) (41)	Singapore	Chronic subcortical stroke	18	tDCS + MI-BCI	MI-BCI	Cathodal tDCS over M1: Intensity 1 mA, 20 min BCI: 40 min, 10 sessions over 2 weeks	FMA-UE, MRI, fMRI
Zhang (2025) (29)	China	Subacute stroke	29	tDCS + KI-BCI	KI-BCI	Cathodal tDCS over M1: Intensity 2 mA,15 min BCI: 15 min, 20 sessions over 4 weeks	FMA-UE, MSS, MBI, EEG
Chew (2020) (42)	Singapore	Chronic stroke	19	tDCS + MI-BCI	MI-BCI	Cathodal tDCS over M1: Intensity 1 mA,20 min BCI: 60 min,10 sessions over 2 weeks	FMA-UE, RMT, SICI, SICF
Jia (2024a) (28)	China	Subacute stroke	14	rTMS + MI-BCI	MI-BCI	Ipsilesional rTMS: Frequency 10 Hz, 100% RMT, Total pulse 600, 20 min BCI: 40trails, 10 sessions over 2 weeks	FMA-UE, ERD
Gao (2022) (43)	China	Chronic stroke	10	rTMS + MI-BCI	MI-BCI	rTMS over M1: Frequency 1 Hz, 80% RMT, Total pulse 1,200, 20 min, 10 sessions over 2 weeks BCI: 30 min,20 sessions over 4 weeks	FMA-UE, DTI
Johnson (2019) (44)	USA	Chronic stroke	3	rTMS + MI-BCI	MI-BCI	rTMS over M1: Frequency 1 Hz, 90%, RMT 10 min BCI:20 trails, 9 sessions over 3 weeks	FMA-UE, MAS, MMSE

BCI, brain-computer interface; MI-BCI, motor imagery-based brain-computer interface; KI-BCI, kinesthetic motor imagery-based brain-computer interface; tDCS, transcranial direct current stimulation; rTMS, repetitive transcranial magnetic stimulation; M1, primary motor cortex; FMA-UE, Fugl-Meyer Assessment for Upper Extremity; MAS, Modified Ashworth Scale; ERD, event-related desynchronization; MRI, magnetic resonance imaging; fMRI, functional magnetic resonance imaging; MSS, Motor Status Scale; MBI, Modified Barthel Index; ARAT, Action Research Arm Test; EEG, electroencephalography; RMT, resting motor threshold; SICI, short-interval intracortical inhibition; SICF, short-interval intracortical facilitation; DTI, diffusion tensor imaging; MMSE, Mini-Mental State Examination.

### Risk of bias and certainty of evidence

Figure 2 summarizes the methodological quality of the included studies assessed using the Physiotherapy Evidence Database (PEDro) scale. Overall, the included studies were of moderate methodological quality, with PEDro scores ranging from 5 to 9. Most studies satisfied the criteria for random allocation, between-group comparisons, and reporting of point estimates and variability. However, methodological limitations were frequently identified in concealed allocation, therapist blinding, and intention-to-treat analysis.

**Figure 2:**
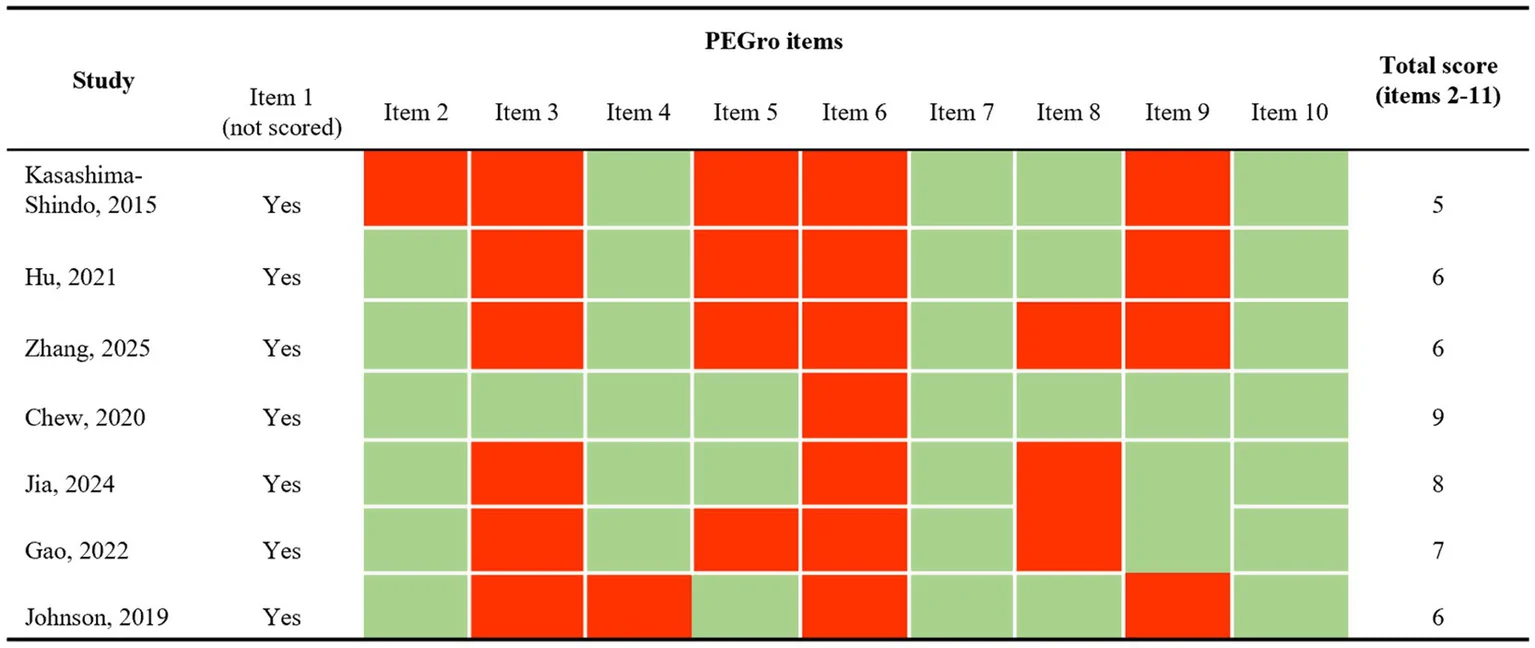
Methodological quality of the included studies assessed using the PEDro scale. Item 1 is not scored. Total scores are based on items 2–11 (maximum score = 10). PEDro items: 1, eligibility criteria; 2, random allocation; 3, concealed allocation; 4, baseline comparability; 5, blinding of subjects; 6, blinding of therapists; 7, blinding of assessors; 8, adequate follow-up; 9, intention-to-treat analysis; 10, between-group comparisons; 11, point estimates and variability.

The certainty of evidence for the effects of combined NIBS and BCI therapy on upper extremity motor outcomes was evaluated using the GRADE approach (Figure 3). For immediate post-intervention outcomes measured by the FMA-UE, the certainty of evidence was rated as moderate in the main analysis (5 studies) and low in the secondary analysis (4 studies). Downgrading was mainly due to risk of bias and imprecision, whereas inconsistency and indirectness were not considered serious. A strong association was observed in both the main and secondary analyses. In contrast, the certainty of evidence for follow-up FMA-UE outcomes (3 studies) and the rTMS subgroup (2 studies) was judged to be very low, reflecting substantial uncertainty in these effect estimates. These ratings were primarily driven by concerns regarding risk of bias and imprecision. Detailed GRADE assessments are provided in Figure 3.

**Figure 3:**
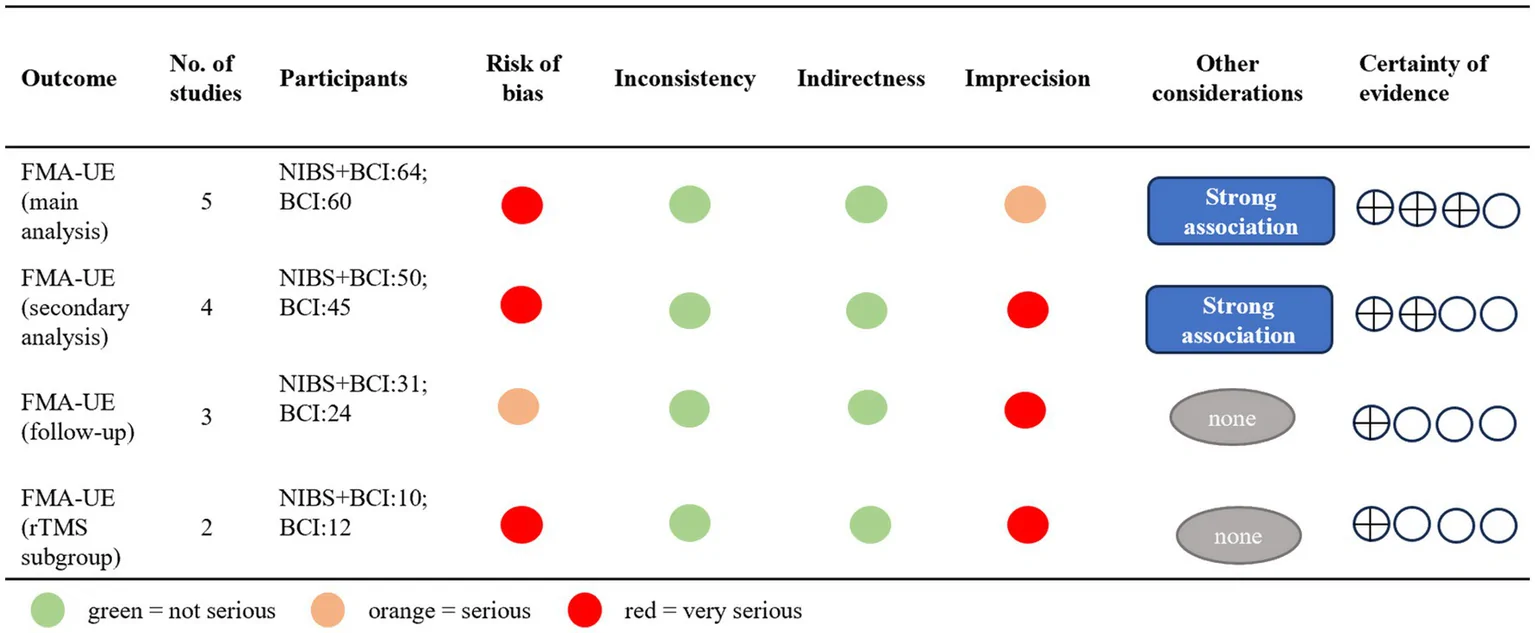
GRADE certainty of evidence for upper extremity motor outcomes.

### Meta-analysis

Five studies involving 124 participants (NIBS+BCI, *n* = 64; BCI alone, *n* = 60) were included in the primary FMA-UE meta-analysis. The pooled analysis showed a significant effect favoring BCI alone over NIBS combined with BCI (MD = −3.67, 95% CI −5.32 to −2.01; I^2^ = 0%; *p* < 0.0001) when all eligible studies, including Zhang et al. (29), were included (Figure 4A). Subgroup analysis indicated that the difference was mainly driven by studies using tDCS combined with BCI (MD = −3.76, 95% CI −5.43 to −2.10; I^2^ = 0%; *p* < 0.00001), whereas no significant difference was observed for rTMS combined with BCI versus BCI alone (MD = 2.65, 95% CI −10.93 to 16.22; I^2^ = 0%; *p* = 0.70). As shown in Figure 4A, Zhang et al. (29) contributed substantial weight to the pooled estimate, particularly within the tDCS subgroup.

**Figure 4:**
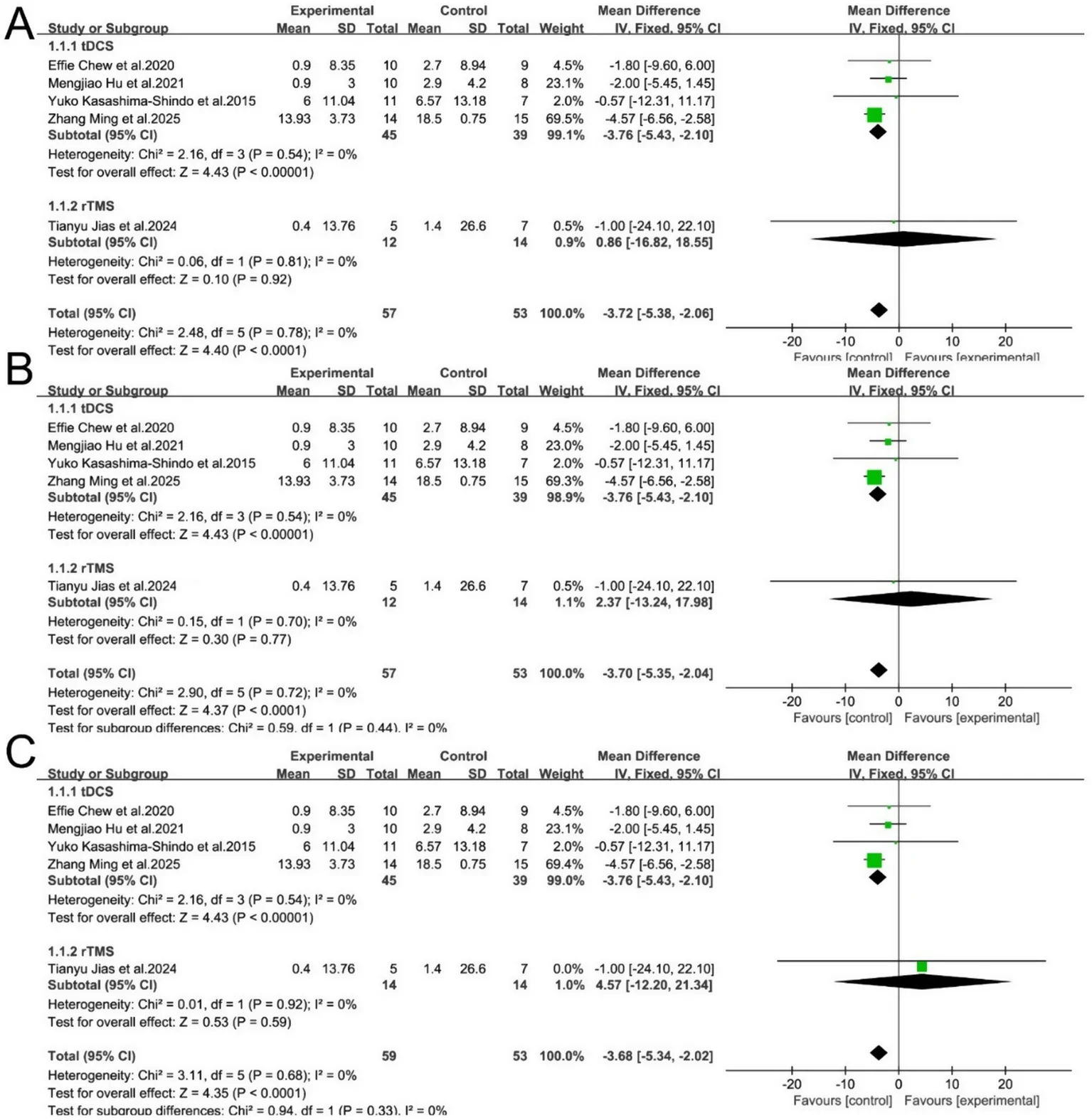
Analysis of the effects of combined non-invasive brain stimulation with brain-computer interface on motor recovery of upper extremity function in post-stroke patients. **(A)** Primary meta-analysis of FMA-UE outcomes including all eligible studies. **(B)** Sensitivity analysis after exclusion of Zhang et al. (29). **(C)** Follow-up meta-analysis of FMA-UE outcomes.

A leave-one-out sensitivity analysis identified Zhang et al. (29) as the most influential study. After exclusion of this study, the overall effect was no longer statistically significant (MD = −1.66, 95% CI −4.63 to 1.32; I^2^ = 0%; *p* = 0.28), and the tDCS subgroup also remained non-significant (MD = −1.87, 95% CI −4.92 to 1.18; I^2^ = 0%; *p* = 0.23) (Figure 4B). Thus, the apparent significant result in the primary analysis was not robust and was largely driven by the inclusion of Zhang et al. (29). Given the unusually small SD reported in the control arm of this study, its disproportionate weight in the pooled analysis should be interpreted cautiously, although the extracted data were consistent with the original report.

Three studies involving 55 participants (tDCS + BCI, *n* = 31; BCI alone, *n* = 24) reported follow-up FMA-UE outcomes. The pooled analysis showed no significant difference between groups (MD = −0.23, 95% CI −4.22 to 3.75; I^2^ = 0%; *p* = 0.91) (Figure 4C). In addition, two studies involving 22 participants compared ipsilesional 1 Hz rTMS combined with BCI versus contralesional 1 Hz rTMS combined with BCI. No significant difference was found between the two stimulation strategies (MD = 0.49, 95% CI −6.38 to 7.37; I^2^ = 0%; *p* = 0.89) (Figure 4).

### Adverse events

No adverse events were reported in the included publications. However, adverse event reporting was inconsistent and likely incomplete across studies, and therefore the absence of reported events should not be interpreted as confirmation that no adverse events occurred.

## Discussion

To our knowledge, this study is the first systematic review and meta-analysis to comprehensively evaluate the clinical efficacy of combining NIBS with BCI training in stroke rehabilitation. Beyond the overall comparison between NIBS plus BCI and BCI alone, we further examined differences according to stimulation modality, including tDCS and rTMS, and explored whether the effects of rTMS combined with BCI varied by stimulation target. Overall, the available evidence does not demonstrate a consistent additive benefit of NIBS when combined with BCI for upper extremity recovery after stroke.

In the primary meta-analysis, NIBS combined with BCI did not demonstrate additional benefit over BCI alone. Subgroup analysis suggested that the direction of the pooled estimate was mainly influenced by studies evaluating tDCS plus BCI, whereas no significant difference was observed between rTMS plus BCI and BCI alone. Although the combination of NIBS and BCI has been hypothesized to produce synergistic effects, such an advantage may not have emerged in the included tDCS studies because the neuromodulatory effects of tDCS are relatively modest and may depend strongly on stimulation parameters, timing, lesion characteristics, and individual responsiveness. In addition, BCI alone may already provide substantial therapeutic benefit, leaving limited room for detecting an incremental effect of adjunctive tDCS. However, these findings should be interpreted with considerable caution. Leave-one-out influence analysis identified Zhang et al. (29) as the most influential study in the primary outcome analysis. This trial contributed a disproportionately large weight to the pooled estimate, and after its exclusion, the between-group difference was no longer statistically significant. In particular, the unusually small SD reported in the control arm of Zhang et al. (29) may have contributed to the disproportionate weight of this study in the pooled analysis. Although the extracted data were consistent with the original report, this feature warrants cautious interpretation. After exclusion of one influential study in the sensitivity analysis, the between-group differences were no longer statistically significant, indicating that the pooled findings were not robust and may have been driven by a single study. Similarly, no significant differences were found in the long-term follow-up analysis of tDCS plus BCI or in the exploratory comparison of different rTMS stimulation targets. Taken together, current evidence does not demonstrate superiority of NIBS combined with BCI over BCI alone, and the observed effect estimates are highly sensitive to individual studies and methodological differences.

The present findings are noteworthy because they do not fully align with the theoretical expectation that combining two neurorehabilitation strategies with putative neuroplastic effects should produce synergistic benefits. Previous meta-analyses have shown that BCI-based interventions can improve upper extremity motor function after stroke, although the magnitude and durability of this effect remain uncertain. In parallel, NIBS techniques such as tDCS and rTMS have been widely investigated as adjunctive tools for modulating cortical excitability and promoting motor recovery (30). Nevertheless, evidence for the efficacy of NIBS alone in stroke rehabilitation has also been mixed, and optimal stimulation protocols remain controversial (31, 32). Therefore, it is perhaps unsurprising that combining NIBS with BCI has not yet yielded a stable and reproducible clinical advantage across studies.

Several factors may explain the absence of a clear additive effect. First, substantial clinical and methodological heterogeneity existed across the included studies. The participants differed in stroke stage, lesion characteristics, baseline motor impairment, and recovery potential, all of which may influence responsiveness to neuromodulation (11, 33). Second, the BCI protocols were not uniform and may have varied in their therapeutic intensity, feedback modality, and underlying neurophysiological mechanisms (11). Third, NIBS parameters differed markedly across studies, including stimulation polarity, frequency, intensity, duration, number of sessions, cortical target, and the temporal relationship between stimulation and BCI training. Because the physiological effects of both tDCS and rTMS are highly parameter-dependent, these variations could substantially affect treatment response and may have obscured potential benefits in pooled analyses (34, 35). The consistently low I^2^ values should therefore be interpreted with caution, as the absence of statistical heterogeneity does not rule out important clinical heterogeneity among the included studies.

The subgroup result indicating that tDCS combined with BCI was less effective than BCI alone should be interpreted with caution. A possible explanation is that the effects of tDCS are highly dependent on stimulation polarity, target site, and its timing in relation to task-specific training. When these parameters are not appropriately tailored to the individual patient’s neurophysiological condition, tDCS may fail to facilitate motor recovery and could potentially interfere with the relearning processes engaged during BCI training (36, 37). In addition, the pooled estimate for this comparison was strongly influenced by Zhang et al. (29), which carried substantial weight in the meta-analysis, possibly in part because of the unusually small SD reported in the control group. However, this explanation remains tentative, particularly because the between-group difference was no longer observed in the sensitivity analysis. Therefore, the available evidence is insufficient to conclude that tDCS undermines the effects of BCI. Instead, the findings suggest that the benefit of combining tDCS with BCI remains uncertain and may be contingent on the design of the stimulation and training protocol (38). Subgroup analyses by NIBS modality and stimulation site were exploratory and should be interpreted cautiously.

By contrast, rTMS combined with BCI did not show a significant advantage over BCI alone, nor did the exploratory analyses identify a clear difference according to stimulation target. This may reflect the complexity of post-stroke cortical reorganization. The optimal target for rTMS likely depends on the stage of recovery, lesion location, hemispheric balance, and the extent of residual corticospinal tract integrity. In some patients, facilitating the ipsilesional hemisphere may be appropriate, whereas in others, suppressing maladaptive contralesional activity may be more beneficial. Such interindividual variability suggests that a “one-size-fits-all” neuromodulation strategy may be inadequate. Future studies should therefore move toward biomarker-informed or personalized stimulation protocols, rather than relying solely on conventional target selection strategies (33, 39).

From a clinical perspective, our findings suggest that the routine addition of NIBS to BCI training cannot yet be recommended on the basis of current evidence. BCI alone may already provide meaningful sensorimotor training and neurofeedback, leaving limited room for further improvement through adjunctive neuromodulation in some settings. It is also possible that any benefit of combined treatment may be confined to specific patient subgroups, such as those defined by stroke chronicity, severity, lesion topology, or neurophysiological profile. Identifying such responder populations should be a priority for future research.

Several limitations of this review should be acknowledged. First, the number of included studies and total sample size were small, limiting statistical power and increasing the risk that pooled estimates were influenced by single studies. Second, the methodological quality of the included trials was generally modest, with only one study rated as excellent on the PEDro scale. PEDro was used because it is commonly applied in rehabilitation research, although it does not provide the same domain-based risk-of-bias assessment as RoB 2. Third, the evidence was downgraded in several analyses because of imprecision and study limitations, reducing confidence in the estimates. Fourth, subgroup analyses and comparisons of stimulation targets were based on a limited number of studies and should therefore be considered exploratory. Finally, publication bias was not formally assessed, because fewer than 10 studies were available for each meta-analysis. The secondary outcome findings were also based on limited data and should be interpreted cautiously.

In summary, this review does not provide convincing evidence that combining NIBS with BCI is superior to BCI alone for improving upper extremity function after stroke. Although the concept of combining neuromodulation with BCI is theoretically appealing, current clinical evidence remains limited, heterogeneous, and methodologically weak. High-quality, adequately powered randomized controlled trials are needed to determine whether specific NIBS modalities, stimulation parameters, treatment timing, or patient subgroups may derive meaningful benefit from this combined approach.

## Conclusion

Current evidence does not demonstrate a clear additional benefit of combining NIBS with BCI training over BCI alone for upper extremity rehabilitation after stroke. The apparent differences observed in some analyses were not robust, and the overall certainty of evidence was low. Therefore, these findings should be interpreted cautiously. These results apply specifically to comparisons with BCI alone and should not be interpreted as evidence of superiority over conventional rehabilitation or usual care. Future high-quality, adequately powered randomized controlled trials are required to determine whether specific stimulation modalities, parameters, or patient subgroups may benefit from this combined intervention.

## Data Availability

The original contributions presented in the study are included in the article/Supplementary material, further inquiries can be directed to the corresponding author.
