# Supplementary material for: Effects of combined non-invasive brain stimulation and brain-computer interface on upper extremity motor function after stroke: a systematic review and meta-analysis
Source: Front Neurol. 2026 Oct 19;17:1993570. doi: 10.3389/fneur.2026.1993570 (PMC13724555; doi:10.3389/fneur.2026.1993570)
Supplement: Supplementary file 1 [file Table_1.docx]

Supplementary Table 1.

| **Pubmed** | | Total |
| --- | --- | --- |
| #1 | (((((((((((((((((non-invasive brain stimulation[Title/Abstract]) OR (NIBS[Title/Abstract])) OR (transcranial electric stimulation[Title/Abstract])) OR ("Transcranial Direct Current Stimulation"[MeSH Terms])) OR (tDCS[Title/Abstract])) OR (transcranial random noise stimulation[Title/Abstract])) OR (tRNS[Title/Abstract])) OR (transcranial alternating current stimulation[Title/Abstract])) OR (tACS[Title/Abstract])) OR ("Transcranial Magnetic Stimulation"[MeSH Terms])) OR (TMS[Title/Abstract])) OR (trigeminal nerve stimulation[Title/Abstract])) OR (TNS[Title/Abstract])) OR (transcutaneous supraorbital nerve stimulation[Title/Abstract])) OR ("Electroconvulsive Therapy"[MeSH Terms])) OR (ECT[Title/Abstract])) OR (transcranial ultrasound stimulation[Title/Abstract])) OR (TUS[Title/Abstract]) | 61335 |
| #2 | (((((((((((((((Brain-Computer Interfaces[MeSH Terms]) OR (Brain Computer Interfaces[Title/Abstract])) OR (Interface, Brain-Computer[Title/Abstract])) OR (Interfaces, Brain-Computer[Title/Abstract])) OR (Brain-Computer Interface[Title/Abstract])) OR (Brain Computer Interface[Title/Abstract])) OR (Brain-Machine Interfaces[Title/Abstract])) OR (Brain-Machine Interface[Title/Abstract])) OR (Interface, Brain-Machine[Title/Abstract])) OR (Interfaces, Brain-Machine[Title/Abstract])) OR (Brain Machine Interface[Title/Abstract])) OR (Brain Machine Interfaces[Title/Abstract])) OR (Interface, Brain Machine[Title/Abstract])) OR (Interfaces, Brain Machine[Title/Abstract])) OR (Machine Interface, Brain[Title/Abstract])) OR (Machine Interfaces, Brain[Title/Abstract]) | 11511 |
| #3 | ((((((((((((((((((((((((((((((Stroke[MeSH Terms]) OR (Strokes[Title/Abstract])) OR (Cerebrovascular Accident[Title/Abstract])) OR (Cerebrovascular Accidents[Title/Abstract])) OR (Cerebral Stroke[Title/Abstract])) OR (Cerebral Strokes[Title/Abstract])) OR (Stroke, Cerebral[Title/Abstract])) OR (Strokes, Cerebral[Title/Abstract])) OR (Cerebrovascular Apoplexy[Title/Abstract])) OR (Apoplexy, Cerebrovascular[Title/Abstract])) OR (Vascular Accident, Brain[Title/Abstract])) OR (Brain Vascular Accident[Title/Abstract])) OR (Brain Vascular Accidents[Title/Abstract])) OR (Vascular Accidents, Brain[Title/Abstract])) OR (Cerebrovascular Stroke[Title/Abstract])) OR (Cerebrovascular Strokes[Title/Abstract])) OR (Stroke, Cerebrovascular[Title/Abstract])) OR (Strokes, Cerebrovascular[Title/Abstract])) OR (Apoplexy[Title/Abstract])) OR (CVA[Title/Abstract])) OR (CVAs[Title/Abstract])) OR (Stroke, Acute[Title/Abstract])) OR (Acute Stroke[Title/Abstract])) OR (Acute Strokes[Title/Abstract])) OR (Strokes, Acute[Title/Abstract])) OR (Cerebrovascular Accident, Acute[Title/Abstract])) OR (Acute Cerebrovascular Accident[Title/Abstract])) OR (Acute Cerebrovascular Accidents[Title/Abstract])) OR (Cerebrovascular Accidents, Acute[Title/Abstract])) OR (Cerebrovascular Accident[Title/Abstract])) OR (Cerebrovascular Accident[Title/Abstract]) | 230392 |
| #4 | #1 AND #2 AND #3 | 42 |

Supplementary Table 2.

| **Web of science** | | Total |
| --- | --- | --- |
| #1 | non-invasive brain stimulation (Topic) or NIBS (Topic) or transcranial electric stimulation (Topic) or transcranial direct current stimulation (Topic) or tDCS (Topic) or transcranial random noise stimulation (Topic) or tRNS (Topic) or transcranial alternating current stimulation (Topic) or tACS (Topic) or transcranial magnetic stimulation (Topic) or TMS (Topic) or trigeminal nerve stimulation (Topic) or TNS (Topic) or transcutaneous supraorbital nerve stimulation (Topic) or electroconvulsive therapy (Topic) or ECT (Topic) or transcranial ultrasound stimulation (Topic) or TUS (Topic) | 155316 |
| #2 | Brain Computer Interfaces (Topic) or Interface, Brain-Computer (Topic) or Interfaces, Brain-Computer (Topic) or Brain-Computer Interface (Topic) or Brain Computer Interface (Topic) or rain-Machine Interfaces (Topic) or Brain-Machine Interface (Topic) or Interface, Brain-Machine (Topic) or Interfaces, Brain-Machine (Topic) or Brain Machine Interface (Topic) or Brain Machine Interfaces (Topic) or Interface, Brain Machine (Topic) or Interfaces, Brain Machine (Topic) or Machine Interface, Brain (Topic) or Machine Interfaces, Brain (Topic) | 53049 |
| #3 | Stroke (Topic) or Strokes (Topic) or Cerebrovascular Accident (Topic) or Cerebrovascular Accidents (Topic) or Cerebral Stroke (Topic) or Cerebral Strokes (Topic) or Stroke, Cerebral (Topic) or Strokes, Cerebral (Topic) or Cerebrovascular Apoplexy (Topic) or Apoplexy, Cerebrovascular (Topic) or Vascular Accident, Brain (Topic) or Brain Vascular Accident (Topic) or Brain Vascular Accidents (Topic) or Vascular Accidents, Brain (Topic) or Cerebrovascular Stroke (Topic) or Cerebrovascular Strokes (Topic) or Stroke, Cerebrovascular (Topic) or Strokes, Cerebrovascular (Topic) or Apoplexy (Topic) or CVA (Topic) or CVAs (Topic) or Stroke, Acute (Topic) or Acute Stroke (Topic) or Acute Strokes (Topic) or Strokes, Acute (Topic) or Cerebrovascular Accident, Acute (Topic) or Acute Cerebrovascular Accident (Topic) or Acute Cerebrovascular Accidents (Topic) or Cerebrovascular Accidents, Acute (Topic) or Cerebrovascular Accident (Topic) or Cerebrovascular Accident (Topic) | 1066781 |
| #4 | #1 AND #2 AND #3 | 293 |

Supplementary Table 3.

| **Cochrane** | | Total |
| --- | --- | --- |
| #1 | MeSH descriptor:Stroke | 92518 |
| #2 | (Strokes):ab,ti,kw OR (Cerebrovascular Accident):ab,ti,kw OR (Cerebrovascular Accidents):ab,ti,kw OR (Cerebral Stroke):ab,ti,kw OR (Cerebral Strokes):ab,ti,kw OR (Stroke, Cerebral):ab,ti,kw OR (Strokes, Cerebral):ab,ti,kw OR (Cerebrovascular Apoplexy):ab,ti,kw OR (Apoplexy, Cerebrovascular):ab,ti,kw OR (Vascular Accident, Brain):ab,ti,kw OR (Brain Vascular Accident):ab,ti,kw OR (Brain Vascular Accidents):ab,ti,kw OR (Vascular Accidents, Brain):ab,ti,kw OR (Cerebrovascular Stroke):ab,ti,kw OR (Cerebrovascular Strokes):ab,ti,kw OR (Stroke, Cerebrovascular):ab,ti,kw OR (Strokes, Cerebrovascular):ab,ti,kw OR (Apoplexy):ab,ti,kw OR (CVA):ab,ti,kw OR (CVAs):ab,ti,kw OR (Stroke, Acute):ab,ti,kw OR (Acute Stroke):ab,ti,kw OR (Acute Strokes):ab,ti,kw OR (Strokes, Acute):ab,ti,kw OR (Cerebrovascular Accident, Acute):ab,ti,kw OR (Acute Cerebrovascular Accident):ab,ti,kw OR (Acute Cerebrovascular Accidents):ab,ti,kw OR (Cerebrovascular Accidents, Acute):ab,ti,kw OR (Cerebrovascular Accident):ab,ti,kw OR (Cerebrovascular Accident):ab,ti,kw | 44078 |
| #3 | #1 OR #2 | 96636 |
| #4 | (non-invasive brain stimulation):ab,ti,kw OR (NIBS):ab,ti,kw OR (transcranial electric stimulation):ab,ti,kw OR (transcranial direct current stimulation):ab,ti,kw OR (tDCS):ab,ti,kw OR (transcranial random noise stimulation):ab,ti,kw OR (tRNS):ab,ti,kw OR (transcranial alternating current stimulation):ab,ti,kw OR (tACS):ab,ti,kw OR (transcranial magnetic stimulation):ab,ti,kw OR (TMS):ab,ti,kw OR (trigeminal nerve stimulation):ab,ti,kw OR (TNS):ab,ti,kw OR (transcutaneous supraorbital nerve stimulation):ab,ti,kw OR (electroconvulsive therapy):ab,ti,kw OR (ECT):ab,ti,kw OR (transcranial ultrasound stimulation):ab,ti,kw OR (TUS):ab,ti,kw | 22178 |
| #5 | (Brain Computer Interfaces):ab,ti,kw OR (Interface, Brain-Computer):ab,ti,kw OR (Interfaces, Brain-Computer):ab,ti,kw OR (Brain-Computer Interface):ab,ti,kw OR (Brain Computer Interface):ab,ti,kw OR (rain-Machine Interfaces):ab,ti,kw OR (Brain-Machine Interface):ab,ti,kw OR (Interface, Brain-Machine):ab,ti,kw OR (Interfaces, Brain-Machine):ab,ti,kw OR (Brain Machine Interface):ab,ti,kw OR (Brain Machine Interfaces):ab,ti,kw OR (Interface, Brain Machine):ab,ti,kw OR (Interfaces, Brain Machine):ab,ti,kw OR (Machine Interface, Brain):ab,ti,kw OR (Machine Interfaces, Brain):ab,ti,kw | 637 |
| #6 | #3 AND #4 AND #5 | 75 |

Supplementary Table 4.

| **Embase** | | Total |
| --- | --- | --- |
| #1 | 'Stroke’/exp OR ‘Strokes’/exp OR ‘Cerebrovascular Accident’/exp OR ‘Cerebrovascular Accidents’/exp OR ‘Cerebral Stroke’/exp OR ‘Cerebral Strokes’/exp OR ‘Stroke, Cerebral’/exp OR ‘Strokes, Cerebral’/exp OR ‘Cerebrovascular Apoplexy’/exp OR ‘Apoplexy, Cerebrovascular’/exp OR ‘Vascular Accident, Brain’/exp OR ‘Brain Vascular Accident’/exp OR ‘Brain Vascular Accidents’/exp OR ‘Vascular Accidents, Brain’/exp OR ‘Cerebrovascular Stroke’/exp OR ‘Cerebrovascular Strokes’/exp OR ‘Stroke, Cerebrovascular’/exp OR ‘Strokes, Cerebrovascular’/exp OR ‘Apoplexy’/exp OR ‘CVA’/exp OR ‘CVAs’/exp OR ‘Stroke, Acute’/exp OR ‘Acute Stroke’/exp OR ‘Acute Strokes’/exp OR ‘Strokes, Acute’/exp OR ‘Cerebrovascular Accident, Acute’/exp OR ‘Acute Cerebrovascular Accident’/exp OR ‘Acute Cerebrovascular Accidents’/exp OR ‘Cerebrovascular Accidents, Acute’/exp OR ‘Cerebrovascular Accident’/exp OR ‘Cerebrovascular Accident'/exp | 542282 |
| #2 | 'non-invasive brain stimulation’/exp OR ‘NIBS’/exp OR ‘transcranial electric stimulation’/exp OR ‘transcranial direct current stimulation’/exp OR ‘tDCS’/exp OR ‘transcranial random noise stimulation’/exp OR ‘tRNS’/exp OR ‘transcranial alternating current stimulation’/exp OR ‘tACS’/exp OR ‘transcranial magnetic stimulation’/exp OR ‘TMS’/exp OR ‘trigeminal nerve stimulation’/exp OR ‘TNS’/exp OR ‘transcutaneous supraorbital nerve stimulation’/exp OR ‘electroconvulsive therapy’/exp OR ‘ECT’/exp OR ‘transcranial ultrasound stimulation’/exp OR ‘TUS’/exp | 95561 |
| #3 | 'Brain Computer Interfaces’/exp OR ‘Interface, Brain-Computer’/exp OR ‘Interfaces, Brain-Computer’/exp OR ‘Brain-Computer Interface’/exp OR ‘Brain Computer Interface’/exp OR ‘rain-Machine Interfaces’/exp OR ‘Brain-Machine Interface’/exp OR ‘Interface, Brain-Machine’/exp OR ‘Interfaces, Brain-Machine’/exp OR ‘Brain Machine Interface’/exp OR ‘Brain Machine Interfaces’/exp OR ‘Interface, Brain Machine’/exp OR ‘Interfaces, Brain Machine’/exp OR ‘Machine Interface, Brain’/exp OR ‘Machine Interfaces, Brain’/exp | 12657 |
| #4 | #1 AND #2 AND #3 | 145 |
